# The nosocomial infection survey among patients suffering from the Coronavirus disease-2019 hospitalized in Ayatollah Rouhani Hospital, Babol

**DOI:** 10.22088/cjim.15.3.509

**Published:** 2024-08-01

**Authors:** Masomeh Bayani, Samaneh Rouhi, Rouzbeh Mohammadi Abandansari, Farzane Jafarian, Zahra Ahmadnia, Hossein Ghorbani, Alireza Firouzjahi, Mohammad Ranaee, Somayeh Ahmadi Goorji

**Affiliations:** 1 Infectious Diseases and Tropical Medicine Research Center, Health Research Institute, Babol University of Medical Sciences, Babol, Iran; 2 Clinical Research Development Unit of Ayatollah Rouhani Hospital, Babol University of Medical Sciences, Babol, Iran; 3 Student Research Committee, Mazandaran University of Medical Sciences, Sari, Iran; 4 Department of Biostatistics, School of Allied Medical Sciences, Shahid Beheshti University of Medical Sciences, Tehran, Iran

**Keywords:** Coronavirus disease-2019, Nosocomial infection, Intensive care unit

## Abstract

**Background:**

Having a weakened immune system can make patients easily get nosocomial infection (NI) with multi-drug resistant (MDR) bacteria and put them in a dangerous situation. It causes long hospital stays, disability, economic burden, and even death. The present study aimed to determine the prevalence of NI in patients suffering from COVID-19.

**Methods:**

In this retrospective study, the information on 250 patients suffering from COVID-19 in the intensive care unit (ICU) (2020 to 2021) was considered. For statistical analysis, analysis of variance (ANOVA), paired samples t-test, and chi-square using SPSS-23 software were used (p<0.05).

**Results:**

Two hundred and fifty hospitalized (107 females and 143 males, mean ± standard deviation (SD) of age; 56.50 ± 17.20) patients were considered. The most (97.60%) medicine prescribed was remdesivir. *Candida* spp*.* (two females), *Escherichia coli* (two females), *Acinetobacter* spp*.* (one female), *Citrobacter* spp*.* (one female), *Pseudomonas* spp*.* (one male), *Sphingomonas* spp*.* (one male), *Stenotrophomonas *spp. (one male) and *Enterobacter *spp*. *(one male) were isolated from the patient’s specimens. Four of seven bacterial isolates were positive for MDR. NI was diagnosed in six patients. There was no significant relationship between the age with the isolated microbes (P=0.154) and MDR (P=0.987) and also between gender with common microbes (P=0.576) and MDR (P=0.143).

**Conclusion:**

The coexistence of bacteria and NI was observed in patients. Remdesivir was prescribed for most patients. Most bacteria were resistant to antibiotics, especially, β-lactams.

Infections that appear about 48 to 72 hours after hospitalization are considered nosocomial infections (NI). The incidence rate of NI in medical centers is 5% to 22% (1). A retrospective review in Belgium in 2020 considered the prevalence rate of NI in 1150 centers in 88 countries in 2017. The results of this research showed that 54% of the patients in the intensive care unit (ICU) represented NI. The mortality rate in patients with proven or suspected NI was 30% (2). A study conducted in Iran in 2020 showed that the rate of NI was less than 2% from 2013 to 2014 (3). A cross-sectional study in 2020 in the North of Iran reported 109 NI cases among 26189 patients (4). In a retrospective cross-sectional study performed in the North of Iran in 2014, it was shown that the most common type of NI was related to *Pseudomonas* spp. (36.84%) and *Acinetobacter* spp. (28.02%) (5). The 2019 -coronavirus disease (COVID-19) is an infectious disease caused by the severe acute respiratory syndrome coronavirus 2 (SARS‑CoV‑2).

The 2019 -coronavirus disease (COVID-19) is an infectious disease caused by the severe acute respiratory syndrome coronavirus 2 (SARS‑CoV‑2). The COVID-19 epidemic severely affected most countries in the world at the beginning of 2020. One of the major challenges imposed by this infection was the increasing number of patients in need of ICU. In 2020, the prevalence rate of NI in patients with COVID-19 was 3.6% to 43% (6). In another study performed in 2020, 22 cases of NI were seen in patients with underlying diseases and immune system disorders​​ affected by COVID-19 (7). On the other hand, the frequent use of antibiotics in patients with COVID-19 causes an increase in antimicrobial resistance and, as a result, the severity of the disease. Among those with bacterial infections, the proportion of infections that were resistant to antimicrobials was 60.8% and the proportion of bacteria isolates that were resistant was 37.5% (8). 

A study reported that co-infection with resistant bacteria or fungi ranged from 0.2% to 100% among COVID-19 patients. Multidrug-resistant bacteria and fungal (methicillin-resistant *Staphylococcus aureus*, carbapenem-resistant *Acinetobacter baumannii*, *Klebsiella pneumoniae*, *Pseudomonas aeruginosa*, and MDR *Candida auris*) were observed among 24% of isolates (9). Infections caused by other microbes following the infection caused by COVID-19 are very dangerous. Microbial identification, outbreak investigation, infection control, and treatment for NI to reach a normal condition in the patient are important and considerable. The results published about the co-existence of NI and COVID-19, especially in the North of Iran are limited. Also, demographic characteristics, health and physical condition, and clinical information of patients in different geographical locations of the world and a country are different in comparison to other locations and affect the severity of the diseases. So, it is very important to pay attention to these variables to diagnose and facilitate the treatment of diseases. The purpose of the present research was to assess the prevalence of NI due to the COVID-19 epidemic and microbes related to NI among patients admitted to the ICU.

## Methods

This study was done in a retrospective; descriptive-analytical way using the information available (Age, gender, medicine prescribed, isolated microorganisms, antibiotic pattern, diseases, the outcome of the disease, and hospitalization period) in the health information system (HIS) designed to manage healthcare data. The data of COVID-19 patients admitted to the ICU were cross-sectional analyzed (from February 2020 to February 2021, Ayatollah Rouhani Hospital, Babol, Mazandaran, Iran). Patients who were not infected with COVID-19 and outpatients with COVID-19 were excluded from the study. The clinical symptoms of patients suspected of having symptoms of COVID-19 were considered by a physician (10). 

The samples used for the laboratory test of COVID-19 include nasopharyngeal swab samples, and sputum or lower respiratory tract aspirate. The diagnostic confirmation test was a real-time polymerase chain reaction (real-time PCR), which identified the ribonucleic acid (RNA) genome of the virus. RNA processing and extraction of the samples were done using the protocols of the viral RNA extraction kit (Roche Diagnostics, Germany). The causative virus of COVID-19 was identified using TaqMan probes with real-time PCR. PCR was performed as follows: a volume of 25 µL contained 12.5 µL of 2xrxn buffer, 10 µM of each primer, 40 μM of primer, 5 pmol probes, 0.5 µL of Rox, 2.5 units of enzyme mix, and 5 µM of RNA template. The real-time PCR reaction was performed in 7300 ABI real-time PCR and the settings were: 50°C for 30 minutes, 95°C for 5 minutes, 40 cycles of denaturation at 95°C for 15 seconds, and annealing and expansion each at 60°C for 30 seconds. Computerized tomography (CT) scan technology and the degree of lung involvement were investigated in the initial diagnosis of the disease. The salient features of chest radiographs in patients with severe pneumonia of COVID-19 include; the view of the glass being opaque and the lungs being dense, which could involve both lungs (11, 12).

A time cut-off of 48 hours after admission was used to distinguish between hospital-acquired and community-acquired infections. NI was identified based on NI criteria as well as infection control nurses in the hospital. Before and at the time of admission, the patient should not show any symptoms of the desired infection and the disease should not be in its latent period. Infection occurs in a limited or widespread form and is a result of pathogenic reactions related to the infectious agent itself or its toxins in the hospital.

It should be established at least 24 hours after the patient is admitted to the hospital. These infections include urinary tract infections, bloodstream infections, surgical site infections, ventilator-associated pneumonia, and hospital-acquired pneumonia. Symptoms that favor an infection include cough, shortness of breath, abdominal pain, rebound tenderness, altered mental status, palpitations, suprapubic pain, polyuria, dysuria, and costovertebral angle tenderness (13). However, colonization means the growth and multiplication of the infectious agent (microorganism) in the host without infection, clinical expression, or immune response (14, 15).

The patient’s specimen was transferred from Stewart's transfer medium on the blood agar, chocolate agar (for general culture), McConkey agar (lactose fermentation), and eosin methylene blue agar (to identify gram-negative bacteria), and mannitol salt agar (to identify bacteria with high salt requirement) (all media were obtained from Merck, Germany). Then mediums were incubated for 24 hours at 37°C. According to the 2018 Clinical and Laboratory Standards Institute (CLSI), the desired antibiotics (colistin (0.16-256 mg/L), ciprofloxacin (5 µg), ceftazidime (30 µg), ceftriaxone (30 µg), amikacin (30 µg), meropenem (10 µg), piperacillin/tazobactam (100/10 µg)) were selected (Padtan Teb, Iran). First, the turbidity bacteria suspension equivalent to 0.5 McFarland standards was prepared and cultured by a sterile swab on Mueller Hinton agar culture medium (Merck, Germany). Second, antibiotic discs were placed on the medium at 2 cm intervals and the plates were incubated at 37 degrees for 24 hours. Finally, the halo of growth inhibition around the antibiotic disk was measured with a ruler and compared and read with the CLSI standard. E-test also was done according to kit structures for colistin (Liofilchem s.r.l, Italy) (16, 17). The germ tube method was used to confirm the presence of *Candia* spp. (18).

All microbial culture tests were performed before antibiotic treatment. The use of antibiotics by patients inhibits the growth of microorganisms on the culture medium. Also, to follow the correctness and treatment process of the infection, to ensure the correct prescription of antibiotics, and to ensure the destruction of the infectious agent, the microbial culture test was repeated according to the physician's request. No positive culture was observed when the patient was treated.

Using G-power software and the following formula, taking into account the first type error of 5% (Z = 1.96) and the assumed percentage of contracting the COVID-19; 50%, the accuracy of the test is 2.6%, the minimum sample size is estimated to be around 250 people (19). Mean and standard deviation (SD), indicators were used to describe quantitative data. Numbers and percentages were used to describe qualitative data, and the Kolmogorov-Smirnnov test was used to check the normality of the data. Analysis of variance (ANOVA) and paired samples t-test were used to check the relationship between quantitative and qualitative variables, and the chi-square method was used to check the relationship between qualitative variables (p<0.05).

## Results

The information on 250 hospitalized patients with COVID-19 (female; 107 (42.80%), male; 143 (57.20%)) was reviewed. The mean ± standard deviation (SD) age of the patients was 56.50 ± 17.20 years (minimum 16 years and maximum 91 years). Most of them were males (57.20%) and over 55 years old (51.60%) with a hospitalization period of less than 14 weeks (86.80%). The mean ± SD days of hospitalization were 8.00 ± 5.80 days. Most patients survived (211 patients; 84.40%). Clinical samples related to 211 (84.40%) patients were cultured on the microbial culture medium. The most prescribed medicines for patients were remdesivir (97.60%), ceftriaxone (91.20%), tucilizumab (90.40%), steroids (88.40%), ciprofloxacin (88.00%), and azithromycin (81.60%) (table 1). 

**Table 1:** Information on variables examined in patients suffering from COVID-19

**Variable**	**Level**	**Prevalence (Number)**	**Prevalence (%)**
**Gender**	Female	107	42.80
	Male	143	57.20
**Hospitalization period**	Less than 14 weeks	217	86.80
	More than 14 weeks	33	13.20
**The outcome of the disease**	Death	39	15.60
	Alive	211	84.40
**Perform microbial culture (yes/no)**	Yes	237	94.80
	No	13	5.20
**Remdesivir**	Yes	244	97.60
	No	0	0.00
**Ceftriaxone**	Yes	228	91.20
	No	22	8.80
**Tucilizumab**	Yes	226	90.40
	No	24	9.60
**Steroids**	Yes	221	88.40
	No	29	11.60
**Ciprofloxacin**	Yes	220	88.00
	No	18	7.20
**Azithromycin**	Yes	204	81.60
	No	46	18.40

Results showed that of 237/250 (94.80%) cultured specimens, 10 (seven females and three males, 4.21%) of them were positive in terms of microbial contamination. The eight of 10 positive microbial cultures were related to bacterial isolates. *Candida* spp*.* (two females; 2.00%), *Escherichia coli* (two females; 2.00%), *Acinetobacter* spp*.* (one female), *Citrobacter* spp*.* (one female), *Pseudomonas* spp*.* (one male), *Sphingomonas* spp*.* (one male), *Stenotrophomonas *spp. (one male) and *Enterobacter *spp*. *(one male) (each one, one case; 1.00%) were detected. MDR was observed in four of eight bacterial isolates. NI was observed in six patients (male and female; each one, three cases. Five cases of deceased patients and one living patient). 

In four of six NI cases, MDR and NI were observed, simultaneously. NI was more common in the age group of > 55 years (five of six NI cases). However, no significant relationship was found between the outcome of patients (alive, deceased) (P=0.286), isolated bacteria (P=0.758), and MDR (P=0.429) with NI. Also, there was no significant relationship between MDR with isolated bacteria (P=0.429) (table 2). 

**Table 2:** Antibiotic resistance pattern of bacterial isolated from patients suffering from COVID-19 and NI

**NI: positive (P)/negative (N**	**MDR: positive (P)/negative (N)**	**Antibiotic Patterns: resistant (R)/sensitive (S)/Intermediate (I)**	**Antibiotics prescribed**	**Type of bacteria**
		S	Colistin	
		R	Ciprofloxacin	
		R	Ceftazidime	
P	P	R	Ceftriaxone	***Citrobacter *** **spp** ***.***
		R	Piperacillin	
		R	Tazobactam	
		R	Amikacin	
		S	Ciprofloxacin	
		S	Amikacin	
P	N	S	Colistin Sulfate	***Sphingomonas*** ** spp.**
		S	Ceftriaxone	
		S	Ceftazidime	
		S	Meropenem	
		R	Ciprofloxacin	
		R	Amikacin	
P	P	R	Piperacillin/Tazobactam	***Pseudomonas*** ** spp.**
		R	Ceftazidime	
		R	Meropenem	
		R	Ciprofloxacin	
		S	Amikacin	
N	N	S	Colistin Sulfate	***Escherichia coli*** ** spp.** ^1^
		S	Piperacillin/Tazobactam	
		R	Ceftriaxone	
		S	Meropenem	
		S	Amikacin	
N	N	S	Colistin Sulfate	***Escherichia coli*** ** spp.** ^2^
		S	Piperacillin/Tazobactam	
		S	Ceftriaxone	
		S	Meropenem	
		S	Amikacin	
		S	Colistin	
		R	Ciprofloxacin	
		R	Ceftriaxone	
P	P	R	Piperacillin/Tazobactam	***Stenotrophomonas*** ** spp.**
		R	Meropenem	
		R	Amikacin	
		S	Colistin	
		R	Ciprofloxacin	
		R	Ceftriaxone	
P	P	R	Piperacillin/Tazobactam	***Acinetobacter*** ** spp.**
		R	Meropenem	
		R	Amikacin	
		S	Colistin	
		R	Ciprofloxacin	
		S	Ceftriaxone	
P	N	S	Ceftazidime	***Enterobacter*** ** spp.**
		S	Amikacin	
		I	Piperacillin/Tazobactam	

**Figure 1:**
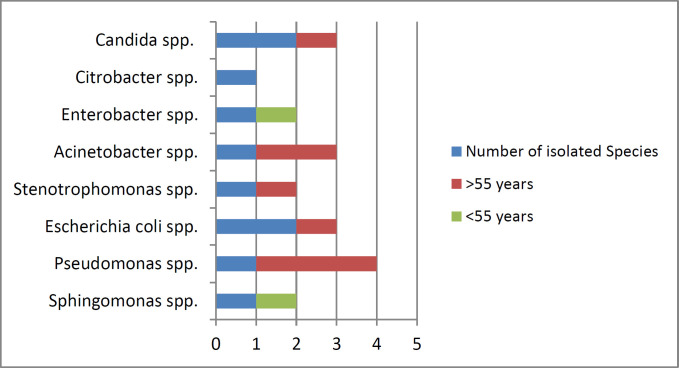
Prevalence of isolated common microbes according to patients' age

**Figure 2:**
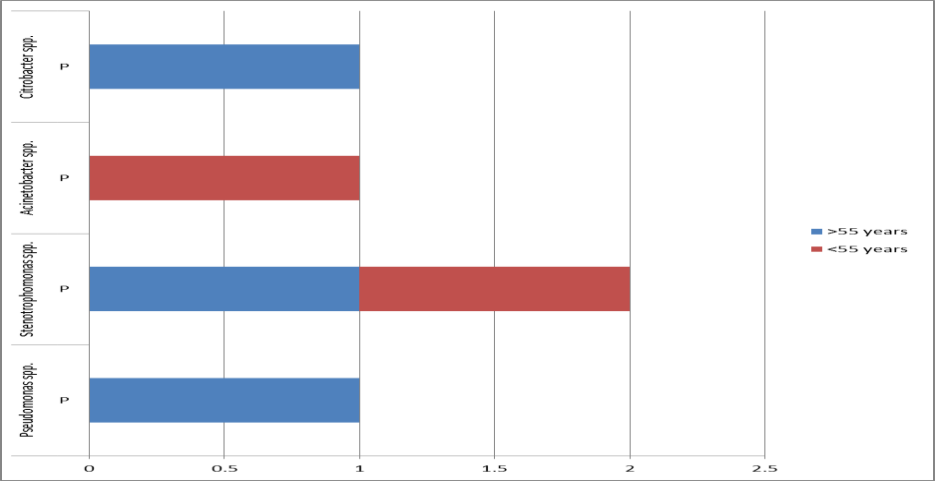
Prevalence of isolated MDR according to patients' age

**Figure 3:**
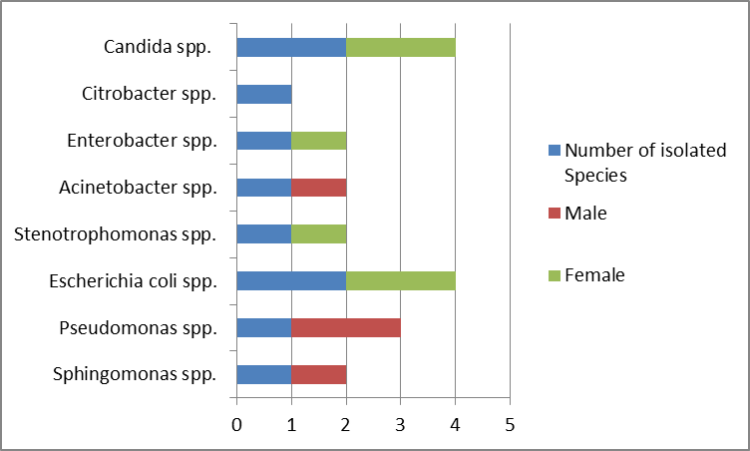
Prevalence of isolated common microbes according to gender

**Figure 4:**
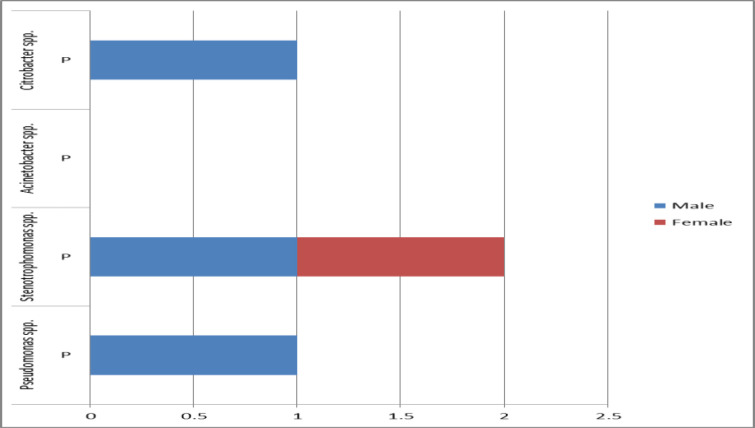
Prevalence of isolated MDR according to gender

The isolated microbes from patients were more common in the age group of >55 years old (eight of 10 cases), but there was no significant relationship between the age with the isolated microbes (P=0.154) (figure 1). Also, the prevalence of MDR (three out of four cases) was higher in the age group of > 55 years old. There was no significant relationship between the age with the MDR (P=0.987) (figure 2). *Candida* (two cases of 10 cases) and *Escherichia coli* (two cases of 10 cases) were observed as the common microbes in female patients. Two (of 10 cases) isolates of *Pseudomonas* spp. were observed in male patients. There was no significant relationship between gender with common microbes (P=0.576) (figure 3). The prevalence of MDR was higher in male patients (three of four cases), but there was no significant relationship between gender with MDR (P=0.143) (figure 4). 

## Discussion

The present research aimed to assess NI and microbe isolates among patient specimens with COVID-19 patients. In this study, the information of 250 hospitalized patients suffering from COVID-19 (mean age; 56.5 years old) was considered. In similar research, Bardi et al. in 2021 in Spain examined 140 patients suffering from severe COVID-19. They were admitted to the ICU between March and May 2020. In the study by Bardi et al., the sampling period was shorter, but the number of patients was more compared to our study (250 patients, 12 months). On the other hand, the sampling in Bardi et al.’s study was done during two months, and the number of 140 patients during two months is also significant (20). We showed that the number of male patients (143 (57.20%)) was more than females (107 (42.80%)). In a similar study performed by Poletti et al. in 2021 in Italy, 2824 confirmed COVID-19 patients (mean age; 53 years old) were considered, and more of them 1604 (56.8%) were females. The symptoms of COVID-19 ranged from 18.10% among patients < 20 years to 64.6% for those >80 years or older (21). Some factors such as the body's immune responses, hormonal and gender characteristics, the severity of illness, and time and place of sampling, can affect the COVID-19 prevalence in different societies (22, 23). In the meanwhile, children have a low risk of catching COVID-19, but they easily transmit infection and are a source of silent SARS-CoV-2 transmission. Elderly people are more likely to get infected with COVID-19 due to weak immune systems and underlying diseases (23, 24).

In our study, the hospitalization period of the more patients (86.60% versus (vs) 13.20% more than 14 weeks) was less than 14 weeks. The average hospitalization days in the ICU were 8 days. 

Also, 94.80% of microbial cultures, showed a positive result of bacteria. Fortunately, 84.40% of patients during our study period survived. In Bardi et al.’s study, the infection occurred after 9 days of hospitalization and 2.95% of patients had positive microbial cultures within 8 days (20). Some risk factors, such as age of more than 70 years, shock, major trauma, coma, prior antibiotics, mechanical ventilation, steroids, chemotherapy, and indwelling catheters can be effective in prolonged hospitalization and the development of NI (25). 

Jabarpour et al. 2021 in Iran reported that the prevalence of patients with COVID-19 was 6135, which was more than our study (250 patients), and the total rate of NI during this period was 3.7%, which had a low rate similar to our study (NI; 6 cases) (26). 

In Bardi et al.'s study, 57 (40.7%) patients had NI (bacterial or fungal infection) during their stay in the ICU, which was higher than our study (6 cases)**. **NI occurs at different rates in patients depending on the patient's condition. NI associated with COVID-19 is significantly associated with death and longer ICU stays (20). Pan et al. in China in 2022 reported that the rate of NI was 7.85% (54/688) in the first period before COVID-19 and 4.30% (26/605) during COVID-19. The incidence of NI decreased during the COVID-19 pandemic (27). However, it seems that the appropriate implementation of infection control protocols during the COVID-19 pandemic appears to reduce NI (26).

We also found that the most prescribed medicine for patients suffering from COVID-19 was remdesivir (97.60%), ceftriaxone (91.20%), tucilizumab (90.40%), steroids (88.40%), ciprofloxacin (88.00%), and azithromycin (81.60%), respectively. 

In the study performed by Michot et al. in France in 2020 (28) and Aziz et al. in 2021 in the United States (USA) (29), tocilizumab was introduced as a monoclonal antibody blocking the interleukin-6 receptor which was applied as an important and effective immunosuppressant in the patients suffering from COVID-19 (28, 29). Delgado et al. in the USA in 2023 found that there was a positive association between the use of remdesivir (an adenosine analog that can inhibit viral replication) by COVID-19 patients in “Late 2020” and reduced odds of mortality (30). 

In another similar study, Mathew and Antony in 2021 in the US reported that tocilizumab could be prescribed as a treatment for patients with COVID-19. In this research, it was shown that; the administration of tocilizumab and antibiotics such as cefepime, azithromycin, ceftriaxone, ciprofloxacin, and piperacillin/tazobactam and steroids reduced the need for invasive mechanical ventilation in patients (31). Using a meta-analysis study, Ayerbe et al. 2022 in the United Kingdom (UK) found that azithromycin as a broad-spectrum antibiotic has antiviral activity, but there was no difference in mortality rate, need for hospital admission, clinical severity, need for ICU, or adverse effects for the patients suffering from COVID-19 treated with or without the use of azithromycin (32).

In our study, *Candida* spp*.*, *Escherichia coli*, *Acinetobacter* spp*.*, *Citrobacter* spp*.*, *Pseudomonas* spp*.*, *Sphingomonas* spp*.*, *Stenotrophomonas *spp*.*, and *Enterobacter* spp*.* were isolated from 10 patient’s specimens. Grasselli et al. in 2021 in Italy reported ventilator-associated pneumonia in patients with COVID-19 caused by Gram-negative bacteria (especially Enterobacteriaceae) (64%) and *Staphylococcus aureus *(28%) (33). 

The study conducted by Yang et al. in 2020 in China showed that 3 of 52 (5.7%) patients with COVID-19 were infected with *Aspergillus flavus*, *Aspergillus fumigatus*, or *Candida albicans* (34). NI is also caused by a wide range of Gram-positive and Gram-negative bacteria, as well as fungi. 

The patients hospitalized in the ICU are sicker than the other patients and may suffer from a lack of calories and protein, they easily get different infections. Artificial pulmonary ventilation and the presence of different venous and urinary catheters have caused microorganisms to colonize and be transferred by nurses and physicians who care for patients and are in contact with them (20, 35). Similar research performed by de Souza et al. in 2023 in Brazil showed that *Pseudomonas aeruginosa, Klebsiella pneumoniae,* and *Acinetobacter baumannii* were the main (68%) MDR- Gram-negative bacteria species isolated from 871 patients with positive COVID-19. de Souza et al. also reported that 22 MDR isolated strains among 70 patients with COVID-19 and NI. In our study, the simultaneous presence of MDR and NI was observed in four of six NI cases. According to the research conducted on NI, it was found that patients in the age range of 60-90 are more exposed to NI. Since these patients may have immune system defects, diabetes, and vitamin deficiency, they are more prone to infection. Children are another group of people who are at risk of getting an infection due to their weak immune system. NI can lead to the death of patients, too (36).

 NI during COVID-19 directly affects the quality of life of patients and also leads to additional costs for hospitals. Healthcare professionals worldwide must form a union against the simultaneous presence of NI and COVID-19. The fight against COVID-19 may provide valuable lessons for the prevention and control of NI in the future (37). Results in our study showed that remdesivir was the main medicine. *Candida* spp*.* and different bacteria species were isolated from patient’s specimens with positive COVID-19. MDR bacteria and NI were observed in patients. There was no significant between patient outcomes, drug resistance, age, and gender with isolated microbes. Also, NI with antibiotic resistance and isolated microbes did not have any significant relationship.

 Our study was a retrospective study that was conducted in a hospital for a limited period and a limited number of samples, so the generalization of our findings to the findings of other similar studies is limited. A large number of patients, whose COVID-19 had led to their rapid death after hospitalization, may not have been registered in the NI system. Also, due to the engagement of physicians and nurses during the COVID-19 pandemic, some accurate statistics on NI have been provided. Therefore, prospective and multicenter studies with a larger number of samples and a detailed study of patients' files are needed. In this regard, meta-analysis studies are suggested for critical evaluation and statistical combination of the results of related studies or trials.
